# Meiotic drive changes sperm precedence patterns in house mice: potential for male alternative mating tactics?

**DOI:** 10.1186/s12862-016-0710-4

**Published:** 2016-06-21

**Authors:** Andreas Sutter, Anna K. Lindholm

**Affiliations:** Department of Evolutionary Biology and Environmental Studies, University of Zurich, Winterthurerstrasse 190, 8057 Zurich, Switzerland

**Keywords:** *t* haplotype, Sperm competition, Selfish genetic element, Multi-level selection, Alternative reproductive tactics, Polyandry, Copulatory behaviour, Ovulation, CASA

## Abstract

**Background:**

With female multiple mating (polyandry), male-male competition extends to after copulation (sperm competition). Males respond to this selective pressure through physiological, morphological and behavioural adaptations. Sperm competitiveness is commonly decreased in heterozygote carriers of male meiotic drivers, selfish genetic elements that manipulate the production of gametes in males. This might give carriers an evolutionary incentive to reduce the risk of sperm competition. Here, we explore this possibility in house mice. Natural populations frequently harbour a well-characterised male driver (*t* haplotype), which is transmitted to 90 % of heterozygous (+/*t*) males’ offspring. Previous research demonstrated strong detrimental effects on sperm competitiveness, and suggested that +/*t* males are particularly disadvantaged against wild type males when first-to-mate. Low paternity success in the first-to-mate role is expected to favour male adaptations that decrease the risk of sperm competition by preventing female remating. Genotype-specific paternity patterns (sperm precedence) could lead to genetically determined alternative reproductive tactics that can spread through gene level selection. Here, we seek confirmation that +/*t* males are generally disadvantaged when first-to-mate and address whether males of different genotypes differ in reproductive tactics (copulatory and morphological) to maximise individual or driver fitness. Finally, we attempt to explain the mechanistic basis for alternative sperm precedence patterns in this species.

**Results:**

We confirmed that +/*t* males are weak sperm competitors when first to mate. When two +/*t* males competed, the second-to-mate was more successful, which contrasts with first male sperm precedence when wild type males competed. However, we found no differences between male genotypes in reproductive behaviour or morphology that were consistent with alternative reproductive tactics.

Sperm of +/+ and +/*t* males differed with respect to *in vitro* sperm features. Premature hypermotility in +/*t* males’ sperm can potentially explain why +/*t* males are very weak sperm competitors when first-to-mate.

**Conclusions:**

Our results demonstrate that meiotic drivers can have strong effects on sperm precedence patterns, and may provide a heritable basis for alternative reproductive tactics motivated by reduced sperm competitiveness. We discuss how experimental and evolutionary constraints may help explain why male genotypes did not show the predicted differences.

**Electronic supplementary material:**

The online version of this article (doi:10.1186/s12862-016-0710-4) contains supplementary material, which is available to authorized users.

## Background

Females of many species mate with multiple males (polyandry), leading to postcopulatory competition between males [1]. With polyandry, a male’s reproductive success is not only determined by his access to mates, but also by how successful his sperm are in competition for fertilisations [2]. Males are predicted to respond to postcopulatory sexual selection through adaptations in ejaculate production and allocation [3, 4]. Alternatively, males may also attempt to reduce the risk of sperm competition by guarding females [1]. The pay-off structure of different male tactics will depend on a number of factors [5, 6]. One of the most important determinants of the pay-off to mate guarding is sperm precedence, the distribution of paternity share among males in sperm competition [5–8]. With last male sperm precedence, i.e., where the last-to-mate male sires the majority of offspring, the potential fitness loss to a first-to-mate male due to sperm competition is larger than with first male precedence. As a consequence, more investment into mate guarding is predicted with last male precedence [6].

Intrinsic variation between males can cause variation in male alternative reproductive tactics (ART). Male ARTs define different ways of intraspecific and intrasexual competition for paternity [9] and typically involve a set of correlated behavioural, physiological and/or morphological traits. The main factors thought to lead to ARTs are differences in the ability to defend females or resources [9]. In fish, large males often follow a bourgeois tactic including mate guarding and parental care, whereas small males with relatively large testes usually follow a parasitic tactic with sneak fertilisations [10]. The possibility that intrinsic variation in sperm competitiveness can cause variation in male reproductive tactics has received little attention. However, Engqvist [11] modelled optimal ejaculate allocation for males that intrinsically vary in sperm competitiveness as a consequence of mitochondrial variation or segregation distorters that act in males. His findings highlight the potential for ARTs as a consequence of intrinsic male variation, with differential allocation between male types especially when intrinsic differences between males are pronounced and polyandry levels are moderate [11].

A potentially wide-spread origin of variation in postcopulatory competitiveness is segregation distortion in males [12]. Meiotic drivers are selfish genetic elements that interfere with fair Mendelian segregation in diploid organisms, and as a consequence are inherited by more than 50 % of the offspring (hence they ‘drive’ [13, 14]). If meiotic drive elements cannot reach fixation, for example due to homozygote lethality, a polymorphism at the drive locus can persist [14]. Drive occurs in heterozygotes, and typically in males [15], the driver kills or interferes with gametes not carrying the driver. As a consequence, male carriers have fewer viable or functional sperm, with important negative consequences for their sperm competitiveness [16, 17]. Empirical evidence supports the notion that male drive commonly reduces sperm competitiveness [12, 18]. Drive elements thus provide a heritable genetic basis for sperm competitiveness, with potential implications for male ARTs. Especially interesting is that fitness of drive-carriers does not have to exceed that of non-carriers for drive-associated ARTs to spread, as fitness accounting takes place at the gene level, and includes the transmission advantage from drive.

The *t* haplotype in house mice is a classic example of male drive that has a long evolutionary history of around 3 million years [19]. Previous research shows that male carriers (denoted as +/*t*) are strongly disadvantaged in sperm competition against wild type (+/+) males [20]. Drive in +/*t* males is due to an elaborate molecular mechanism comparable to a “poison-antidote” system that results in abnormal flagellar function of + sperm within a +/*t* ejaculate [21]. At least four distorters (the “poison”) and the responder (the “antidote”) are part of the *t* haplotype’s large set of linked genes that are protected from recombination by four major inversions that take up about one third of chromosome 17 [21]. Gamete interference within the +/*t* ejaculate results in the majority (typically around 90 %) of the offspring of a +/*t* male inheriting the *t*, while transmission follows the fair rules of mendelian inheritance in female carriers. An important aspect of the sperm competition findings is that +/*t* males obtain a very small paternity success when competing against wild type males, indicating that the “poison-antidote” system of the *t* haplotype leaves *t* sperm partially impaired [20]. Curiously, there is no order effect in +/*t* versus +/+ sperm competition, contrasting with the first male sperm precedence previously described for house mice [22, 23], and suggesting that +/*t* males may not be able to benefit from the usual first-to-mate advantage [20]. Given their very weak sperm competitiveness, +/*t* males might follow a reproductive tactic where they attempt to secure paternity by preventing sperm competition. Female house mice have been shown to be actively polyandrous in the lab [24, 25], and multiple paternity is common in wild-caught females [26–28]. A strong disadvantage particularly in the defensive (i.e., first-to-mate) sperm competition role should strengthen +/*t* males’ incentive to prevent female remating with other males. Depending on the efficacy of prevention of female remating, an increased effort by +/*t* males could compensate for the disadvantage and result in equal fitness for both genotypes, or alternatively, +/*t* males could be doing the “best of a bad job” [29]. Interestingly, the same argument can be applied at the gene level, where fitness might be the same for the *t* haplotype and its wild type counterpart, or the *t* haplotype could be doing the best of a bad job. Differences in behaviour between +/*t* and +/+ mice in other contexts demonstrate the *t* haplotype’s potential to influence behavioural traits, although there is limited consistency across studies: genotypes may differ with respect to female preference ([30]; but see [25]), male social dominance (+/*t* > +/+ [31] and +/*t* < +/+ [32]), and female personality and life-history strategy [33].

Here, we investigate in house mice whether +/*t* males are indeed generally disadvantaged in defensive sperm competition and how that might affect male reproductive tactics. The pay-offs of alternative tactics can strongly depend on sperm precedence patterns [6]. First, we compare the paternity outcome from sperm competition between two +/*t* males to sperm competition between two +/+ males. We then explore the possibility of alternative reproductive tactics in males by measuring a suite of behavioural and morphological traits related to reproduction. Male house mice might have a variety of possibilities to influence the risk of sperm competition. Later ejaculation relative to oestrus stage may benefit +/*t* males when they are first to mate by reducing the time available for (and thus the likelihood of) female remating [34]. Similarly, extended copulatory stimulation may reduce sperm competition risk by reducing female receptivity to other males [35–37], and repeated ejaculation provides a paternity advantage in mice [20, 38]. Large copulatory plugs produced by proteins from the seminal vesicle and coagulating gland can delay female remating and increase paternity share of first-to-mate males [38]. Plugs thus offer some potential to increase reproductive success through passive mate guarding [39, 40]. Alternatively, investing into scent marking to signal social dominance and territory ownership [41] and to attract females [42] via proteins from the preputial gland may increase reproductive success [43]. We address the possibility of alternative reproductive tactics in +/*t* and +/+ males by observing copulatory behaviour and assessing investment into different male reproductive organs that account for the production of ejaculate components and scent marks. Finally, we attempt to mechanistically link the sperm precedence patterns to sperm phenotypes by assessing temporal dynamics of sperm features *in vitro*.

## Methods

### Experimental animals

Study subjects were male and female wild house mice (*Mus musculus domesticus*) that were laboratory-born F1 to F3 descendants from a free-living population near Illnau, Switzerland [44], from which we introduce wild-caught individuals into our breeding colony every generation. We bred and kept mice under standard laboratory conditions under a 14L:10D cycle (breeding colony: lights on at 05:30 CET; mating experiments: reversed cycle with lights on at 17:30 CET) at a temperature of 22–24 °C with food (laboratory animal diet for mice and rats, no. 3430, Kliba) and water provided *ad libitum*, and paper towels and cardboard served as enrichment and nest building material. Our laboratory population is derived from a wild population that harboured a single *t* haplotype variant with strong male drive and homozygote lethality [45]. Breeding pairs consisted of monogamous pairs of non-sibling +/+ males and either +/+ or +/*t* females, the latter producing on average 50 % +/*t* offspring. At the age of 23 days, we weaned offspring, took a tissue sample by ear punch for genotyping and individual identification, and kept them in same sex sibling groups in Makrolon Type III cages (23.5 × 39 × 15 cm). We used +/*t* and +/+ males and females and diagnosed their *t* haplotype status before they entered the experiment. DNA extraction was performed by salt-chloroform extraction [46] and *t* haplotype status was diagnosed as described elsewhere [45, 47]. Male mice were separated at latest at the onset of aggression between brothers and kept individually in Makrolon Type II cages (18 × 24 × 14 cm). Mice were moved from the breeding colony room into the experimental room at least 2 weeks before being used in the experiment to allow for acclimatisation to the reversed light cycle. The experimenter was blind with respect to genotype during all procedures, including mating trials, female and male dissections, sperm analyses, and video observations (see below).

### Sperm competition trials

For this study, we made use of sperm competition trials from an experiment on the effect of copulatory plugs on rival (second-to-mate) male behaviour and paternity outcome [38]. We used both +/*t* and +/+ males and females, focusing on competition between brothers of the same *t* genotype for paternity data. Two full brothers from the same litter competed against each other in order to control for potential effects of genetic background and maternal environment on sperm competitiveness. For behavioural analyses, we focus on first-to-mate males and how their copulatory behaviour may relate to reducing the risk of sperm competition. Mating trials were conducted as specified elsewhere [38]. Briefly, a sexually receptive female [48] was introduced into a male’s cage. Trials were started 2.5 h ± 0.5 (mean ± SD) after the beginning of the dark phase, and females were subsequently checked for a copulatory plug (indicating ejaculation [49]) every 1–1.5 h. Once a plug was detected, the trial was stopped and the plug was either removed or left intact [38], after which the female was paired with the second male and checked every 30–60 min until either a new copulatory plug was observed or until the beginning of the next dark phase. After the second mating, plugs were again removed or left intact, and mated females were kept in isolation. Females that did not mate were re-tested on a later occasion. We used a paired design for our plug removal treatment [38], so that males were used in multiple mating trials.

### Paternity assignment

To get paternity estimates that were unbiased by embryonic mortality associated with homozygous effects of the *t* haplotype [20, 50], we sacrificed females 9 days *post coitum* using gradual CO_2_ filling in their home cage and recovered embryos under a dissection microscope at 10–40x magnification. Paternity was assigned using the software CERVUS [51] on genotypes from 12 microsatellites spread across ten autosomes, with details as described elsewhere [20].

### Alternative reproductive tactics?

#### Copulatory behaviour

We used video recordings to obtain detailed information on copulatory behaviour of both males. The first male’s first ejaculation would sometimes go undetected during a trial, when the male dislodged his own plug after ejaculation but before the female was checked for the presence of a plug. For paternity analyses, we recorded the number and timing of both males’ ejaculations, as reported previously [38]. Here, we additionally recorded details on first-to-mate males. From the first copulatory series, we recorded (i) the latency from introduction to the first mount, (ii) the number and (iii) average duration of copulatory bouts (mounts and mounts with intromission), (iv) the latency from the first copulatory mount to ejaculation, and (v) the *in copula* duration at ejaculation. As a proxy for the male’s motivation to repeatedly mate with the same female, we also assessed (vi) the latency from ejaculation to the initiation of a second copulatory series (post-ejaculation interval).

#### Ejaculation timing relative to ovulation

To investigate potential differences between male genotypes in their ejaculation timing relative to ovulation, we used the extent of cornification of epithelial cells in vaginal smears as a proxy for female oestrus stage [34, 48]. We took vaginal smears using plastic inoculation loops and took digital photographs under a microscope at 100x magnification. Images were scored by a single observer for the proportion of cornified epithelial cells at steps of 0.1. Oestrous stage scores from 50 pictures assessed independently on two different days showed high intra-observer repeatability for scoring (F_49,50_ = 13.1, *p* < 0.001, *R* = 0.928).

#### Male reproductive organs

Here, we investigated whether weak sperm competitors (+/*t* males) invest differently into traits important for pre- versus postcopulatory selection than strong sperm competitors (+/+ males). For all males involved in mating experiments and sacrificed for sperm analyses (see below), we measured the relative organ weights of the preputial glands (pheromone production), testes and the entire epididymides (sperm production and storage), and seminal vesicles and coagulating glands (copulatory plug production; dissected pairwise).

### Sperm features

To investigate effects of the *t* haplotype on sperm features, we compared sperm features of +/+ and +/*t* males *in vitro*. Full details of the procedures are provided as supplementary methods (Additional file 1). Briefly, we analysed sperm of 12 pairs of sexually mature +/*t* and +/+ brothers from monogamous breeding pairs. Males were kept in isolation and were sacrificed using gradual CO_2_ filling in their home cage. The order of dissection was randomised and all procedures were done blind. We dissected both caudal epididymides and incubated sperm in modified human tubal fluid (mHTF; Bühlmann Laboratories AG) at 37 °C. Using computer assisted sperm analysis (CASA; MouseTraxx, Hamilton Thorne), we measured patterns of sperm velocity and linearity (average path velocity VAP, straight-line velocity VSL, curvilinear velocity VCL, amplitude of lateral head displacement ALH, beat cross frequency BCF, straightness STR, and linearity LIN). Repeated measurements over a large time span have been recommended for obtaining data on both initial swimming speed and the rate of decline [52]. We attempted to cover the time period that sperm are stored *in vivo* between ejaculation and ovulation, which has previously been estimated at between 2 and 5 h in a monogamous context in laboratory mice [53, 54]. Thus, for every male we measured a large number of sperm paths (mean ± SD = 327 ± 270) at each of 4–5 time points after different incubation times between 15 min and 6 h.

### Statistical analyses

An overview of the sample sizes available for the different analyses is given in Table 1. The data set supporting the results of this article is available in the Dryad repository, doi:10.5061/dryad.m2h55. Using the functions lmer and glmer in lme4 [55] in R version 3.1.3 [56], we analysed data on paternity outcome, sperm features, copulatory behaviour and reproductive organs with either linear mixed models (LMM), or generalised mixed models (GLMM). We extracted effect sizes from full models to avoid biasing effect sizes through removal of non-significant terms [57]. To test the global null hypothesis, we compared full models to null models using likelihood ratio tests [57]. For LMMs, we obtained *p*-values for fixed effects using *F*-tests between full models and a model excluding the factor of interest, with degrees of freedom based on the Kenward-Roger approximation implemented in the package pbkrtest [58]. To improve interpretability, some continuous input variables were standardised to a mean of 0 and a standard deviation of 1 (see Table 2) as recommended by Schielzeth [59]. We calculated approximate 95 % confidence intervals (c.i.) by multiplying Student’s *t*-values for our sample sizes by standard errors of the predicted values [60].

**Table 1:** Overview of sample sizes available for the different analyses

		Male genotype combination			
Subsection	Sample sizes	+/*t* vs +/*t*	+/*t* vs +/*+*	+/*+* vs +/*t*	+/+ vs +/*+*
Sperm precedence	N mating trials (N embryos)	17 (117)	–	–	23 (179)
Copulatory behaviour	N mating trials (N different individual males)	17 (10)	10 (4)	9 (4)	39 (14)
		Male genotype			
		+/*t*		+/*+*	
Male reproductive organs	N males	40		48	
Sperm features	N males	12		12

**Table 2:** Full model summaries for P_2_ and sperm features

Model	Response variable	Random effects	Fixed effects	Mean (SD)	Fixed effect centred/ standardised?	Estimate [approx. 95 % c.i.]	*z* value/ *F* value	*p*
GLMM	P_2_:	Male ID	Intercept (competition +/+ vs +/+)			**−1.47 [−2.39, -0.54]**	−3.21	**0.001**
	Paternity share 2^nd^ male		Male genotype combination (+/*t* vs +/*t*)	–	n/n	**4.30 [2.55, 6.04]**	4.99	**< 0.001**
			Ejaculation number 1^st^ male – 2^nd^ male	0.3 (0.5)	n/n	**−1.39 [−2.75, −0.04]**	−2.08	**0.038**
			Ejaculation interval 1^st^ to 2^nd^ male [h]	2.0 (0.9)	y/y	**−1.39 [−2.12, −0.67]**	−3.88	**< 0.001**
			Weight difference 1^st^ male – 2^nd^ male	−1.3 (2.2)	y/y	0.22 [**−**0.42, 0.86]	0.70	0.484
			Female genotype (28 +/+; 12 +/t)	–	y/n	1.33 [**−**0.03, 2.69]	1.96	**0.048**
LMM	PC1:	Brother pair/Male ID	Intercept (centred for genotype)			**0.32 [0.15, 0.48]**	–	–
	Progressive sperm speed	Incubation time*Male ID	*t* haplotype	–	y/n	**−0.35 [−0.66, −0.04]**	4.44	**0.048**
			Incubation time [h]	2.5 (2.0)	n/n	**−0.05 [−0.07, −0.02]**	9.80	**0.005**
			Sperm count	71.3 (36.7)	y/y	**−0.28 [−0.38, −0.17]**	23.60	**< 0.001**
			*t* haplotype*Time	–	–	0.01 [**−**0.05, 0.06]	0.04	0.850
			*t* haplotype*Sperm count	–	–	0.05 [**−**0.08, 0.18]	0.43	0.517
			Sperm count*Time	–	–	0.01 [**−**0.02, 0.03]	0.40	0.528
LMM	PC2:	Brother pair/Male ID	Intercept (centred for genotype)			**0.27 [0.08, 0.47]**	–	–
	Sperm head speed	Incubation time*Male ID	*t* haplotype	–	y/n	0.12 [**−**0.18, 0.41]	0.53	0.476
			Incubation time [h]	2.5 (2.0)	n/n	**−0.10 [−0.13, −0.08]**	48.10	**< 0.001**
			Sperm count	71.3 (36.7)	y/y	−0.09 [**−**0.22, 0.03]	1.75	0.190
			*t* haplotype*Time	–	–	−0.06 [**−**0.11, 0.003]	3.07	0.096
			*t* haplotype*Sperm count	–	–	0.10 [**−**0.06, 0.27]	1.17	0.285
			Sperm count*Time	–	–	0.005 [**−**0.03, 0.04]	0.06	0.809

*LMM* linear mixed model, *GLMM* generalised linear mixed model. Intercepts correspond to estimates for wild type (+/+) males (paternity share) or were centered for male genotype (sperm features) by assigning values of -0.5 and +0.5 to +/+ and +/*t* males, respectively. Thus, intercepts in the sperm models correspond to an average between +/+ and +/*t* males for an incubation time of 0 h, and *t haplotype* shows the change in sperm features for +/*t* relative to +/+ males. Approximate 95 % confidence intervals were obtained by multiplying Student’s *t*-values for our sample sizes by standard errors of the predicted values [60]. 95 % confidence intervals not overlapping zero and *p* values < 0.05 are highlighted in bold. Degrees of freedom for *F* values were based on the Kenward-Roger approximation [58]

#### P_2_

We analysed the proportion of embryos sired by the second male (P_2_) with binomial GLMMs. The number of embryos sired by the second male was included as the dependent variable and the number of offspring genotyped as the binomial denominator. To investigate sperm precedence patterns in relation to the genotype combination of competing males, we ran a GLMM on P_2_, with the full model including the following variables: male genotype combination (factor with two levels), the body weight difference of the two males, the difference in ejaculation numbers of the males, the interval between both males’ first ejaculations, and female genotype. Male identity was included as a random effect to avoid pseudoreplication. Dispersion parameters of the GLMMs were ≈ 1.

#### Copulatory behaviour

Our recorded variables were not sufficiently correlated to justify a reduction of dimensionality. Thus, we analysed the components of copulatory behaviour individually using LMMs. Full models contained male and female body weight, and male and female genotype as fixed effects. To investigate whether males adjust the timing of their ejaculation to female oestrus stage, we included the proportion of cornified cells (our measure of oestrus stage) and its interaction with male genotype as additional covariables. We included male identity as a random effect to avoid pseudoreplication. Full models were compared to null (intercept-only) models using likelihood ratio tests on the global null hypothesis that the focal behaviour was unaffected by any of the included fixed effects [57].

Because post-ejaculation interval included many (30/83) right-censored data points (when trials were discontinued after detection of a plug and the male had not yet performed any post-ejaculatory mounts), we analysed it with a cox proportional hazard model in the survival package [61].

#### Male reproductive organs

The weights of preputial glands, testes, epididymides, and seminal vesicles and coagulating glands were analysed using LMMs with brother pair as a random effect to account for similarity caused by relatedness and shared early environment. As fixed effects we included male body weight, male genotype and their interaction term.

#### Sperm features

We measured sperm traits from 25,284 individual sperm in 828 scans at 4–5 different time points for each for 24 males. Mean values per sperm sample may be a poor representation of a sample’s fertilisation potential or competitiveness, given that most sperm will not make it to the fertilisation site [52]. In the context of the *t* haplotype, the drive mechanism reduces the fertilisation potential of a large proportion of a +/*t* male’s sperm. Moreover, in our *in vitro* measurements, a considerable proportion of the measured sperm stuck to the cover slide (see [62] for how to avoid this problem). Indeed, many of our sperm variables showed a bimodal distribution, most likely as a result of having both stuck and free swimming sperm in our samples. For all these reasons, we subset our dataset to include only the upper 50 % per sample, based on curvilinear velocity (since this velocity measure is least affected by the shape of sperm movement). Sperm traits were correlated and were reduced using principal components analysis (PCA) using the function *principal* in the psych package [63]. Both Bartlett’s and Steiger’s tests clearly rejected the null hypothesis that all correlations between traits were zero (see Additional file 1: Table S3), the Kaiser-Meyer-Olkin measure of sampling adequacy was moderate at 0.57 (calling for a cautious interpretation), and parallel analysis suggested that extracting two components was adequate. Components were rotated using the orthogonal varimax method and scores were calculated using regression. We then averaged scores to obtain a single value for a given male at a given incubation time (*N* = 104) for each component, and the two components were then analysed using LMMs. The full models contained the male’s genotype, incubation time, the number of sperm counted (averaged across replicate scans; range = 20–176, corresponding to 1.3–12.8 million sperm/mL) and all two-way interactions as fixed effects. Since we had repeated measures and a sibling design, we included individual-specific random intercepts (nested within male brother pair) and individual-specific random slopes for incubation time to avoid overconfidence in interaction estimates [64]. The percentage of motile sperm was averaged across replicate scans and was analysed separately.

## Results

### Sperm precedence

**Fig. 1:**
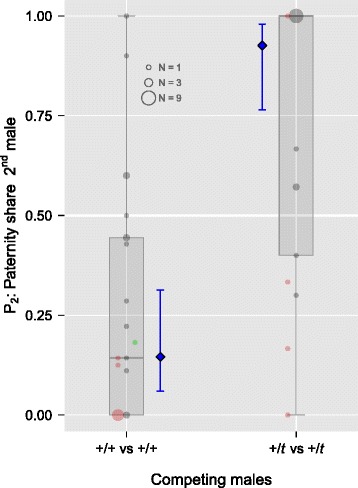
Sperm precedence patterns change with male genotype combination. Shown is the paternity share of the second-to-mate male (P_2_). P_2_ was below 0.5 (first male precedence) when two +/+ males competed, but above (second male precedence) when two +/*t* males competed. *Boxplots* and *circles* show the raw data, with area size corresponding to the number of observations. *Red circles* depict sperm competition trials in which the first male ejaculated twice; the *green circle* shows a trial with two ejaculations by the second male. *Blue diamonds* and error bars show the model predictions and 95 % confidence intervals from a GLMM accounting for other fixed effects (see main text)

In a controlled sperm competition experiment, we mated female house mice consecutively to two different males. We analysed paternity data from sperm competition trials in which two +/*t* males or two +/+ males had competed, and successfully assigned paternity for 311 of the 332 embryos dissected from 42 pregnant females. The paternity share of the second male to mate (P_2_) ranged from zero to one, with many incidences of exclusive paternity for one of the males (48 %) despite multiple mating. P_2_ varied strongly with the combination of male genotypes. Mean P_2_ was 0.27 when two +/+ males competed, but rose to 0.72 when two +/*t* brothers competed (raw data in Fig. 1). We then investigated in more detail which factors determined paternity success, incorporating behavioural data on timing and number of ejaculations. A full model on 40 trials with complete information (Table 1; *N* = 23 for +/+ vs +/+; *N* = 17 for +/*t* vs +/*t*) showed significant effects of the *t* haplotype (versus wild type), the difference in the number of ejaculations, the interval between the two males’ ejaculations, and female genotype (Table 2). Repeated ejaculation by the first male decreased P_2_ (*z* =−2.31, *p* = 0.038), as did a longer delay between the first and the second males’ ejaculation (*z* =−3.88, *p* < 0.001). P_2_ was higher in +/*t* females (*z* = 1.96, *p* = 0.048). After controlling for other factors, mean P_2_ was predicted at 0.15 [95 % c.i. = 0.06, 0.31] for competition between two +/+ males and 0.93 [0.76, 0.98] for competition between two +/*t* males, respectively (Fig. 1, model predictions). Thus, both P_2_ predictions were highly significantly different from equal paternity share, but showed an inversion from first male sperm precedence when two +/+ males competed to second male precedence when two +/*t* males competed. We combined paternity success data in a payoff matrix to compare individual level and gene level success, taking into account the transmission advantage of the *t* haplotype (Table 3). Comparing the relative fitness at the gene level, the *t* haplotype has a maximum of 0.23 of the fitness of its wild type counterpart in sperm competition when first-to-mate but 1.88 when second-to-mate, and 1.8 without sperm competition. Rival male genotype does not strongly influence these pay-offs.

**Table 3:** Pay-off matrix for +/+ and +/*t* males for different mating scenarios, and relative fitness for +/*t* males and the *t* haplotype

	Monandry	Polyandry			
		First-to-mate		Second-to-mate	
	No rival	+/+ rival	+/*t* rival	+/+ rival	+/*t* rival
Paternity share focal +/+ male	1	0.85	0.89	0.15	0.89
Paternity share focal +/*t* male	1	0.11	0.07	0.11	0.93
**Relative fitness +/** ***t*** **male**	**1**	**0.13**	**0.08**	**0.73**	**1.04**
**Relative fitness** ***t*** **haplotype**	**1.8**	**0.23**	**0.14**	**1.32**	**1.88**

Paternity estimates are based on GLMM model predictions for a scenario where both males ejaculate once and the interval between the first and second male’s ejaculation as well as female genotype are centered (see results). Paternity shares for sperm competition between +/+ and +/*t* males are taken from Sutter and Lindholm [20]. Relative fitness (indicated in bold) is expressed for the +/*t* male (the *t* haplotype), with the fitness of the +/+ male (the *t* haplotype’s + counterpart) set to one. Relative fitness of the *t* haplotype thus combines paternity share with segregation distortion. Transmission of the *t* from +/*t* males was assumed at 0.9 as estimated for this laboratory population elsewhere [45]

### Alternative reproductive tactics?

#### Copulatory behaviour

**Fig. 2:**
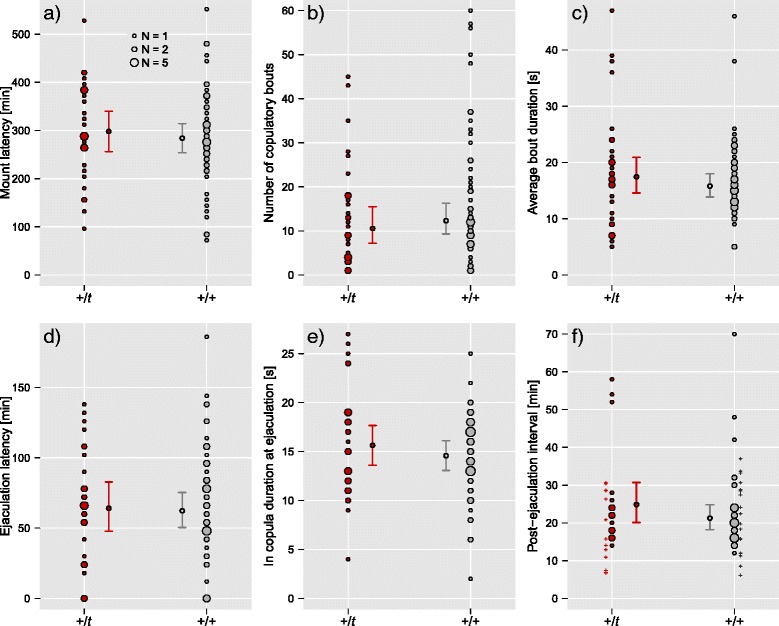
Copulatory behaviour of first-to-mate males. Shown is variation in six different aspects of copulatory behaviour of +*/t* and +/+ males: **a** mount latency, **b** the number of copulatory bouts, **c** average duration of copulatory bouts, **d** ejaculation latency, **e** in copula duration at ejaculation, and **f** post-ejaculation interval. Right-censored data for post-ejaculation interval are indicated with plus symbols (i.e. minimum times for males that were separated from the female before performing post-ejaculatory mounts; see main text). None of the behaviours showed a significant association with male genotype at the *t* locus (see main text and Additional file 1: Table S1). +/*t* males are shown in *red*, +/+ males in *grey*. Points and error bars depict model predictions and approximate 95 % confidence intervals obtained from full models (LMMs, back-transformed to the original scale where necessary)

From our sperm competition trials, we recorded detailed copulatory behaviour for 83 first-to-mate males. Figure 2 shows variation in copulatory behaviour in relation to male genotype at the *t* locus. Summary statistics for copulatory behaviour of +/*t* and +/+ males are given in Additional file 1: Table S1 of the ESM. Comparisons of full models for the different aspects of copulatory behaviour to their respective null models revealed that the global null hypotheses could not be rejected (Additional file 1: Table S1). Thus, neither mount latency, the number or average duration of copulatory bouts (log transformed), latency to ejaculation (sqrt transformed) and *in copula* duration at ejaculation showed any strong evidence for an association with male or female genotype or body weight, or with oestrus stage (Full model tests: all *p* > 0.08; *N* = 75 trials with complete information). The only association between behaviour and a phenotypic or genotypic variable was that heavier males had a shorter ejaculation latency (F_1,37_ = 5.20, *p* = 0.028), though this became non-significant when accounting for multiple testing (*p* = 0.096). Univariate analyses on the effect of male genotype did not support any influence of the *t* haplotype on copulatory behaviour (Additional file 1: Table S1; all *p* > 0.254; *N* = 83).

In addition, we used data on post-ejaculation interval from 53 trials (19 of which involved +/*t* males), complemented with right-censored data from 30 trials (13 trials with +/*t* males) to ask whether +/*t* and +/+ males showed different behaviour. The cox proportional hazard model met the proportional hazards assumptions and indicated no difference between +/*t* and +/+ males (exp(*ß*) = 1.18, *p* = 0.573).

#### Male reproductive organs

There was no difference between the body weight of +/*t* and +/+ males (mean ± SD: +/*t* males 26.3 ± 2.0 g; +/+ males 26.5 ± 2.4 g; F_1,71_ = 0.73, *p* = 0.396). The weights of preputial glands (log transformed), testes and epididymides were correlated with body weight (preputial: F_1,69_ = 27.74, *p* < 0.001; testes: F_1,82_ = 14.46, *p* < 0.001; epididymides: F_1,78_ = 32.40, *p* < 0.001) but showed no significant differences between +/*t* and +/+ males (preputial: F_1,85_ = 1.06, *p* = 0.307; testes: F_1,79_ = 0.71, *p* = 0.403; epididymides: F_1,82_ = 3.08, *p* = 0.083). Seminal vesicles and coagulating glands (weighed pairwise) also correlated positively with body weight (F_1,77_ = 35.22, *p* < 0.001) and showed a significant difference between +/*t* and +/+ males (F_1,68_ = 5.27, *p* = 0.025). Thus, +/+ males had heavier seminal vesicles and coagulating glands relative to body weight than +/*t* males (predicted mean difference [95 % c.i.] = 11.6 mg [2.0, 21.2] = 6 % [1 %, 11 %] of the total weight). The interaction between male body weight and genotype was not significant for any of the organs (all *p* > 0.161).

### Sperm features

We obtained measurements of features of sperm that had left the epididymis during 10 min initial incubation. We repeatedly measured these samples 4–5 times each over several hours of *in vitro* incubation for 24 males (12 +/+ and +/*t* full brothers). Summary statistics of sperm features for +/+ and +/*t* males for different time periods are given in Additional file 1: Table S4. We analysed sperm features from a PCA on 121 ± 93 sperm from each of 4–5 time points for 24 males. The two extracted principal components are summarised in Additional file 1: Table S2 and the correlation matrix is given in Additional file 1: Table S3. The first component (PC1) explained 49.5 % of the variation in sperm features and was positively loaded by measures of path straightness and linearity (STR, LIN) and the smoothed and linear speed (VAP, VSL). The second component (PC2) explained 26.9 % of the variation and was positively loaded by the speed and displacement of the sperm head (VCL, ALH) and by the smoothed path velocity (VAP). Thus, males with higher PC1 scores had linear and progressive sperm, whereas males with higher PC2 scores had sperm whose heads moved vigorously. A combination of low PC1 values and high PC2 values is an indication for hypermotility (see Discussion), vigorous nonlinear movement triggered during activation of mammalian sperm.

**Fig. 3:**
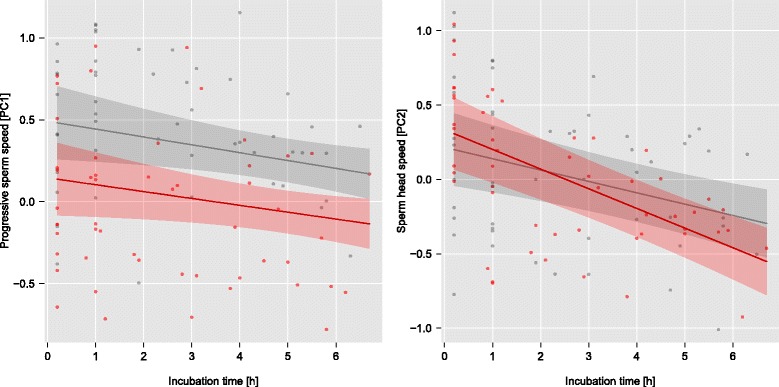
Temporal dynamics of *in vitro* progressive sperm speed (PC1; *left panel*), and sperm head speed (PC2; *right panel*). Raw data are shown as *red* (+/*t* males) and *grey circles* (+/+ males). Lines correspond to predictions from full models (random slope LMMs) including interaction terms and centred for sperm count. Shaded areas depict approximate 95 % confidence intervals. The interaction between *t* haplotype genotype and incubation time was not significant for PC1 (*p* = 0.850) and tended to be negative for PC2 (*p* = 0.096; see main text and Table 2). Thus, sperm linearity and progressiveness (PC1) decreased over time for both male genotypes. In contrast, sperm head speed (PC2) tended to decrease more strongly for +/*t* than for +/+ males (*right panel*). For principal component loadings, see Additional file 1: Table S2

Full model results for both components are shown in Table 2 and illustrated in Fig. 3. Progressive sperm speed (PC1) was lower for +/*t* than for +/+ males (main effect *b* [95 % c.i.] =−0.35 [−0.66,−0.04] for an incubation time of zero and centred for sperm numbers; F_1,19_ = 4.44, *p* = 0.048). Progressive speed also decreased over time and with higher sperm density, but there were no significant interactions (all *p* > 0.5). Sperm head speed (PC2) decreased over time, but tended to do so faster for +/*t* than for +/+ males (interaction genotype x incubation time−0.06 [−0.11, 0.003]; F_1,19_ = 3.07, *p* = 0.096). Sperm count did not have any significant effect on PC2.

Sperm count did not change over time (F_1,21_ = 0.02, *p* = 0.877), but tended to be higher for +/*t* males (F_1,11_ = 3.47, *p* = 0.088). The percentage of motile sperm tended to be initially higher for +/*t* than for +/+ males (F_1,11_ = 4.52, *p* = 0.056). Additionally, there was a trend for an interaction between male genotype and incubation time (F_1,21_ = 3.44, *p* = 0.078). Thus, the higher percentage of motile sperm of +/*t* males tended to increase over time, whereas for +/+ males it decreased slightly.

## Discussion

In controlled sperm competition trials, we confirmed that +/*t* males and the *t* haplotype are highly disadvantaged in the first-to-mate role, which is in strong contrast with the first male precedence when wild type male house mice compete. We expected that this genetically determined sperm precedence inversion would have favoured the evolution of differences in male copulatory or morphological traits that could correspond to differences in the incentive for preventing female remating. However, we did not find differences between male genotypes in male copulatory behaviour, body mass, and reproductive tissues that were consistent with our expectations. In investigating the mechanistic basis of sperm precedence inversion, we found that the *t* haplotype decreased linear sperm velocity and showed signs of premature hypermotility, but had no significant adverse effect on sperm numbers or motility.

### The *t* haplotype inverses sperm precedence

Sperm precedence strongly depended on the competing males’ genotype at the *t* locus. When wild type brothers competed, we found the first male advantage (model prediction for P_2_ = 0.15) that is typical for house mice [22, 23]. However, when females were mated to two +/*t* brothers, second males obtained the majority of the paternity share (predicted P_2_ = 0.93). By including detailed observations on the number and timing of ejaculations, we were able to largely rule out the possibility that this sperm precedence reversal was an experimental artefact. In a previous experiment we had shown that when accounting for the number of ejaculations, there was no order effect in sperm competition between +/*t* and +/+ males [20]. Our current experiment confirmed that +/*t* males are drastically disadvantaged when first-to-mate irrespective of the genotype of the second-to-mate male.

Males carrying driving elements are commonly disadvantaged in sperm competition against wild type males [12, 20]. Price et al. [65] showed that *Drosophila pseudoobscura* males carrying a driving X chromosome obtained a very small paternity share when second-to-mate (P_2_ = 0.14) instead of the typical second male sperm precedence (P_2_ ≈ 0.8 [66]). When first-to-mate, they performed similarly to wild type males (P_1_ = 0.35 [65]). Intriguingly, sperm precedence in *D. pseudoobscura* changes to extreme first male precedence when sperm are stored for long time periods [67]. Other species with male drive show variable patterns. Driver males are equally disadvantaged in both mating roles in *Drosophila simulans* [68] and the stalk-eyed fly *Teleopsis whitei* [69]. These examples demonstrate that more species need to be investigated to identify common effects of male drive on sperm precedence.

### Consequences for male reproductive tactics?

Over the long evolutionary history of the *t* haplotype [19], the genetically determined difference in defensive sperm competitiveness between +/+ and +/*t* males could have led to genetically determined alternative reproductive tactics. Sperm precedence patterns are predicted to strongly influence the pay-off of mate guarding, with last male precedence generally favouring male mate guarding [5–8]. For example, Sherman [70] concluded that mate guarding appeared evolutionarily stable in a ground squirrel species with last male precedence, whereas resuming searching for additional females after copulation was inferred as the stable strategy for another ground squirrel species with first male precedence [70]. Given the strong difference between sperm competitiveness in the defensive mating role, the pay-offs of male tactics to reduce or prevent female remating should differ drastically between +/+ and +/*t* males. Thus, +/*t* males should have a strong evolutionary incentive to prevent female remating, because of the large paternity loss. In contrast, +/+ males are strong defensive sperm competitors, and consequently have little to lose after inseminating a previously unmated female. Table 3 illustrates how with imperfect prevention of female remating, +/*t* males can only make the best of a bad job. However, when considering fitness at the gene level, partially efficient prevention of female remating could lead to equal fitness between the *t* haplotype and its + counterpart when the transmission advantage balances the paternity loss due to sperm competition. Another potential consequence of the combination of sperm precedence patterns and transmission distortion is that the *t* haplotype might create a higher incentive for males to mate with previously mated females (Table 3). However, the pay-offs in this scenario will depend more strongly on the rival male genotype and whether the female will mate with yet another male, and the fitness benefit is limited to *t*-linked genes. Moreover, we have previously reported that copulatory behaviour did not differ between +/*t* and +/+ males when second-to-mate [38].

In order to explore the possibility of male alternative reproductive tactics related to the *t* haplotype, we investigated a variety of behavioural and morphological traits within our experimental setting. We hypothesised that prolonged copulation or repeated ejaculation with the same female could serve as a form of mate guarding [34, 36, 71], and consequently, that +/*t* males would attempt to prolong copulation or to reduce postejaculatory interval compared to +/+ males. Additionally, +/*t* males’ ejaculates may be more competitive closer to the time of ovulation and thus later in oestrus (see below). The difference in sperm competitiveness between +/*t* and +/+ ejaculates could also result in the two genotypes experiencing different pay-offs from resource allocation towards sperm versus alternative fitness-enhancing features [11]. Here, we indirectly assessed male investment into scent marking, sperm production and copulatory plug production by measuring the weights of preputial glands, testes and epididymides, and seminal vesicles and coagulating glands. Collectively, we found no evidence for different reproductive tactics in +/*t* and +/+ males. The only trait that showed a significant difference between +/*t* and +/+ males was the weight of seminal vesicles and coagulating glands, but the lower weight in +/*t* males was opposite to what we had predicted based on the involvement of copulatory plug size in passive mate guarding [38]. Males are limited in seminal fluids when ejaculating repeatedly [72], but whether +/*t* males become limited more quickly as a function of smaller glands is currently unknown.

Several factors may explain why we did not find any of the hypothesised adaptations to low sperm competitiveness in +/*t* males. First, our experimental setting may not have reflected a setting in which males exhibit their different tactics. The behavioural traits we measured are likely to be highly phenotypically plastic and males may have behaved simply in accordance with the experimental conditions. For example, preferential allocation to mate acquisition and retention may only be expressed when directly interacting with other males, where +/*t* males may invest more into suppressing competitors [73]. However, previous research in semi-natural settings has produced contrasting results, with +/*t* males being either more [31] or less [32] socially dominant. With regards to the morphological features measured, differences in resource allocation along trade-offs between pre- and postcopulatory traits may only be discovered when resources are limited [74]. Second, the efficacy of mate guarding is a strong determinant for male tactic pay-offs [7]. If females benefit from polyandry, sexual conflict over remating may prevent efficient mate guarding [75, 76]. The *t* haplotype present in our population is, like many other *t* haplotypes [77], associated with embryonic lethal effects, resulting in strong genetic incompatibility between *t* heterozygous mating partners [45]. Polyandry can strongly reduce the cost of this genetic incompatibility [20]. Thus, sexual conflict might limit the possibility for +/*t* males to prevent female remating. P_2_ was slightly higher for +/*t* than for +/+ females, potentially indicating that +/*t* females discriminate in general against first-to-mate males. However, the biological meaning of this is unclear. Here, both mates had the same genotype at the *t* locus and there was thus no fitness benefit to biasing P_2_. Moreover, we found no evidence for cryptic female choice in sperm competition trials involving +/+ versus +/*t* males [20]. More experiments are needed to elucidate the influence of female choice on the *t* haplotype [25, 30, 45]. Similarly to conflict between the sexes, constraints arising from male-male competition might affect ejaculation timing. Delaying ejaculation relative to ovulation may be too risky for +/*t* males under the threat of a take-over by a rival. Male mice respond to the proximity of a rival by premature ejaculation, possibly an adaptation to the risk of take-overs in natural contexts [34]. We kept all experimental mice in the same room, and thus olfactory and auditory cues may have created a perceived risk of take-over. Third, a model on ejaculate expenditure predicts that the adaptive difference between intrinsically subfertile males and strong sperm competitors not only depends on the difference in sperm competitiveness between the males, but is also sensitive to the frequency of subfertile males and the level of polyandry [11]. House mice show strong temporal and spatial variation in density [78], with potential consequences for variation in polyandry levels [26] and *t* haplotype frequencies [79, 80]. This means that optimal resource allocation becomes a moving target, and that selection should favour phenotypic plasticity rather than fixed tactics for +/*t* and +/+ males [81]. Our study suggests that the pay-off to first-to-mate males does not depend on the frequency of +/*t* males, since P_1_ is largely independent of the rival male’s genotype. In a natural population, the pay-offs of different tactics will likely depend on a number of additional factors such as the adult sex ratio, female mating rates and male mating capacity, the frequency of males employing a mate guarding tactic, and male control over female remating [39, 40]. Combining theoretical models with more empirical data from natural populations would be needed to address the evolutionary plausibility of our predictions more quantitatively. Fourth, the investigated traits – copulatory behaviour and resource allocation to reproductive organs – are likely highly polygenic. As such, the *t* haplotype may exert only limited control. As highlighted above, despite the old evolutionary age of the *t* haplotype [19], temporal and spatial variation may have prevented the stability in selection required to build-up epistatic interactions between the *t* haplotype and the many non-linked genes underlying these polygenic traits. The genetic architecture underlying the traits under selection may impose strong constraints on the evolution of alternative phenotypes [82].

### Can sperm characteristics explain sperm precedence?

We investigated sperm features over an extended period of *in vitro* incubation in an attempt to find a proximate explanation for the disadvantage of +/*t* males in defensive sperm competition. Our sperm measurements showed that sperm movement patterns differed significantly between the two genotypes. Sperm from +/*t* males showed lower progressive speed (smaller PC1 values) over the whole incubation period investigated. In contrast, sperm head speed was initially not different between +/*t* and +/+ males, but tended to decrease faster for +/*t* males (*p* = 0.096 for the interaction between *t* haplotype and incubation time). Previous studies have shown a decrease in progressiveness using *t* haplotypes that had been introgressed into laboratory strain backgrounds (reviewed in [83]). Furthermore, the negative effect of the *t* haplotype on average progressiveness without affecting average initial head speed that we found here is in line with several studies that have found premature hypermotility in sperm from +/*t* males measured as *in vitro* movement and ova penetration [84–86], *in vivo* sperm movement and transport [87, 88], and indirectly from a higher metabolic rate [89]. Hypermotility is characterised by high curvilinear velocity and low straight-line velocity [90], roughly corresponding to high PC2 values and low PC1 values as found for +/*t* males in the early phases of *in vitro* incubation.

The tendency for +/*t* males to have larger numbers of sperm and higher proportions of motile sperm (Additional file 1: Table S4) than +/+ males may be related to our incubation method and the premature hypermotility exhibited by sperm from +/*t* males. Before incubation, epididymides were cut gently and sperm were required to swim out into the medium within 10 min. Our method may thus have selected sperm with more vigorous and non-linear movement. Also, the medium we used represented a benign environment that can sustain high sperm motility over time [91]. Natural ejaculates can behave quite differently from epididymal sperm [62], and *in vivo* conditions impose strong selection on ejaculate quality [92].

*In vivo*, the premature hypermotility in +/*t* ejaculates is a likely candidate for the weak sperm competitiveness of +/*t* males and the sperm precedence inversion found in our experiment. In mammals, activation results in hypermotile sperm, which is crucial for fertilisation and is usually triggered in the oviduct [93]. In hamsters and lemurs, there is evidence for an optimal insemination timing relative to ovulation [94, 95]. Our data indicate that the optimal timing may additionally depend on the male’s ejaculate features. We hypothesise that wild type ejaculate features are co-adapted with insemination timing such as to maximise fertilisation efficacy, and that +/*t* ejaculates deviate from those features. Prematurely hyperactivated sperm in +/*t* ejaculates may over time fall below a minimum threshold movement required to penetrate the ova vestments [96]. This may become most relevant for first-to-mate males when the time window between insemination and fertilisation is substantial. The interval between coitus and ovulation in laboratory mice is estimated at between 2 and 5 h [53, 54], and females ovulate towards the beginning of the light phase [54]. In our experiment, first-to-mate males ejaculated at 7.3 h ± 1.5 (mean ± SD) into the 14 h dark phase, followed by second males 2.0 h ± 0.8 later. If +/*t* ejaculates are deficient in maintaining their fertilising potential over the time period between ejaculation and release of ova, this could explain why +/*t* males obtain a particularly low paternity share in a defensive sperm competition role. Future experiments could experimentally manipulate the timing of ejaculation relative to ovulation and the interval between two rivals’ ejaculations to confirm or refine this hypothesis.

## Conclusions

Using experimental sperm competition trials, our study confirms previous findings that +/*t* males are particularly weak sperm competitors when mating in the first-to-mate role typically favoured in mice. The effects of drive elements on sperm precedence patterns and their transmission advantage highlight their potential for influencing male reproductive tactics [11]. However, our data on copulatory behaviour and reproductive organs did not support alternative reproductive tactics that would have matched our predictions based on sperm precedence patterns. We show that sperm precedence patterns in house mice change from a first male advantage in wild type males to a second male advantage when two *t* haplotype carrying males compete. Sperm from +/*t* males show marked differences in their swimming patterns compared to sperm from +/+ males, and may fall below a velocity threshold during short-term storage between insemination and fertilisation.

## Additional file

Additional file 1: Supplementary results and material and methods. (DOC 90 kb)
