# Supplementary material for: Meiotic drive changes sperm precedence patterns in house mice: potential for male alternative mating tactics?
Source: BMC Evol Biol. 2016 Jun 21;16:133. doi: 10.1186/s12862-016-0710-4 (PMC4915163; doi:10.1186/s12862-016-0710-4)
Supplement: Additional file 1: — Supplementary results and material and methods. (DOC 90 kb) [file 12862_2016_710_MOESM1_ESM.doc]

**Electronic Supplementary Material (ESM):**

**Meiotic drive changes sperm precedence patterns in house mice: potential for male alternative mating tactics?**

*Andreas Sutter and Anna K. Lindholm*

Department of Evolutionary Biology and Environmental Studies, University of Zurich, Winterthurerstrasse 190, 8057 Zurich, Switzerland

Author for correspondence: Andreas Sutter (email: [andreas.sutter@ieu.uzh.ch](mailto:gerald.heckel@iee.unibe.ch))

**Supplementary Material and Methods:**

*Computer assisted sperm analysis (CASA)*

We used full siblings from the same litter to account for potential environmental and genetic effects other than the *t* haplotype on sperm features. Sperm of brothers were measured on the same day to account for potential temporal variation in methodology and measurement instruments and the order of males within a brother pair was randomised. We used 24 sexually mature males (+/+ and +/*t* littermates) at 10-14 weeks of age. Males were different from the ones used in the sperm competition experiment and were either sexually naïve (14 males) or had been given up to eight mating opportunities and had mated between one and four times as part of a different experiment (10 males). Males with sexual experience were sexually rested for at least a week before their sperm were analysed. We sacrificed males using gradual CO_2_ filling in their home cage, and dissected and weighed both caudae epididymides. We pooled both caudae and made multiple incisions with fine scissors in a 1mL drop of pre-warmed modified human tubal fluid (mHTF; Bühlmann Laboratories AG) immersed under mineral oil. Samples were incubated in 37 °C, and epididymal tissue was removed after 10 min. From each sample, 4-5 measurements were taken at different incubation times along a time series (at 15 min, 1 h, and at 2-6 h). We used computer assisted sperm analysis (CASA; MouseTraxx, Hamilton Thorne) to quantify sperm motility parameters. Approximately 7μl of the sperm suspension was loaded into both chambers of a prewarmed improved Neubauer hemocytometer with a chamber depth of 100µm and scanned at 100x magnification. We performed four replicate scans from both chambers of the hemocytometer (one exception: only one chamber measured) for every sample at each time point, so that 327 ± 270 (mean ± SD) motile sperm were recorded for every sample per time point. The sperm suspension was diluted 1:1 with 50μl of pre-warmed mHTF if sperm concentration was determined too high by the CASA system. We used the default mouse settings with minor adjustments (30 frames at 60 Hz; minimum contrast 50, minimum cell size 8 pixels) and recorded average path velocity (VAP), straight-line velocity (VSL), curvilinear velocity (VCL), amplitude of lateral head displacement (ALH), beat cross frequency (BCF), straightness (STR), and linearity (LIN). Individual files with track details were generated for every male at every time point.

**Supplementary Results:**

*Copulatory behaviour*

**Table S1:** Copulatory behaviour of first-to-mate males, their variability indices for +/*t* and +/+ males, p-values (p_full_) from likelihood ratio tests on the null hypothesis that behaviour was unaffected by any of the fixed effects (male and female *t* haplotype genotype and weight, oestrus stage and its interaction with male genotype), as well as p-values from univariate models on copulatory behaviour as a function of male genotype (p_geno_). The p-value for post-ejaculation interval was obtained from a cox proportional hazard model that included right-censored data (see main text).

| **Behavioural variable** | **Mean ± SD** | | **p_full_** | **p_geno_** |
| --- | --- | --- | --- | --- |
|  | +/*t* | +/+ | N = 75 | N = 83 |
| Time to first mount (mount latency) [min] | 299 ± 97 | 286 ± 103 | 0.843 | 0.456 |
| Number of copulatory bouts | 13.3 ± 11.7 | 18.3 ± 15.4 | 0.085 | 0.254 |
| Average duration of copulatory bouts [s] | 18.4 ± 10.1 | 17.2 ± 7.0 | 0.811 | 0.479 |
| Latency to ejaculation [min] | 65.2 ± 38.1 | 70.2 ± 40.3 | 0.081 | 0.567 |
| *In copula* duration at ejaculation [s] | 15.8 ± 5.4 | 14.5 ± 4.3 | 0.842 | 0.355 |
| Post-ejaculation interval [min] | 26.1 ± 13.3 | 23.4 ± 11.2 | – | 0.576 |

*Principal component analysis of sperm features*

**Table S2:** Recorded sperm features (subset of upper 50% sperm based on curvilinear velocity), their variability indices and results from a principal component analysis (PCA). The number of extracted components was determined using parallel analysis. Components were rotated using the varimax method and scores were calculated using regression. Variable loadings of more than 0.4 were considered interpretable and are highlighted in bold [1].

| ***Sperm feature*** | ***Mean*** | ***SD*** | ***PC1*** | ***PC2*** |
| --- | --- | --- | --- | --- |
| Average path velocity (VAP) | 143.8 | 43.2 | **0.737** | **0.523** |
| Straight-line velocity (VSL) | 105.4 | 54.1 | **0.953** | 0.270 |
| Curvilinear velocity (VCL) | 262.6 | 60.4 | 0.331 | **0.839** |
| Amplitude of lateral head displacement (ALH) | 12.8 | 3.7 | -0.346 | **0.828** |
| Beat cross frequency (BCF) | 23.7 | 12.9 | -0.113 | -0.378 |
| Straightness (STR) | 69.6 | 23.7 | **0.910** | 0.037 |
| Linearity (LIN) | 39.4 | 18.1 | **0.972** | -0.066 |
| Sums of squares of loadings | – | – | 3.46 | 1.89 |
| Proportion of variance explained | – | – | 49.5% | 26.9% |

**Table S3:** Correlation matrix for the sperm features included in the principal components analysis. Sperm measurements were based on 12’614 sperm from 828 scans at 4-5 time points for each of 24 males.

|  | VAP | VSL | VCL | ALH | BCF | STR | LIN |
| --- | --- | --- | --- | --- | --- | --- | --- |
| VAP | 1 | 0.849 | 0.744 | 0.073 | -0.127 | 0.523 | 0.650 |
| VSL | 0.849 | 1 | 0.551 | -0.099 | -0.154 | 0.866 | 0.901 |
| VCL | 0.744 | 0.551 | 1 | 0.460 | -0.105 | 0.278 | 0.184 |
| ALH | 0.073 | -0.099 | 0.460 | 1 | -0.258 | -0.165 | -0.315 |
| BCF | -0.127 | -0.154 | -0.105 | -0.258 | 1 | -0.203 | -0.156 |
| STR | 0.523 | 0.866 | 0.278 | -0.165 | -0.203 | 1 | 0.909 |
| LIN | 0.650 | 0.901 | 0.184 | -0.315 | -0.156 | 0.909 | 1 |

VAP = Average path velocity; VSL = Straight-line velocity; VCL = Curvilinear velocity; ALH = Average lateral head displacement; BCF = Beat cross frequency; STR = Straightness; LIN = Linearity

**Table S4:** Summary statistics of mean sperm features of +/*t* and +/+ males over incubation time. Displayed are genotypic means and their standard errors. For any given incubation time, the means of the upper 50% (based on VCL) of motile sperm per individual male were averaged across 4-12 males, depending on incubation time. For % motile and sperm count, means from 8 scans per male were averaged across males.

| Genotype | Incubation [h] | VAP | VSL | VCL | ALH | BCF | STR | LIN | % Motile | Count |
| --- | --- | --- | --- | --- | --- | --- | --- | --- | --- | --- |
| +/+ | 0.25 | 166 ± 8 | 134 ± 8 | 293 ± 13 | 13 ± 0.4 | 33 ± 0.9 | 76 ± 2 | 44 ± 2 | 46 ± 5 | 65 ± 10 |
|  | 1 | 177 ± 6 | 148 ± 7 | 292 ± 9 | 12 ± 0.3 | 33 ± 0.9 | 81 ± 2 | 51 ± 2 | 46 ± 5 | 53 ± 7 |
|  | 2 | 154 ± 17 | 126 ± 19 | 261 ± 19 | 11 ± 0.3 | 33 ± 1.4 | 76 ± 6 | 46 ± 5 | 37 ± 12 | 53 ± 10 |
|  | 3 | 165 ± 6 | 136 ± 8 | 283 ± 11 | 12 ± 0.4 | 32 ± 1.4 | 81 ± 2 | 48 ± 2 | 49 ± 8 | 58 ± 16 |
|  | 4 | 163 ± 8 | 137 ± 9 | 277 ± 6 | 12 ± 0.3 | 28 ± 1.3 | 82 ± 2 | 50 ± 3 | 49 ± 8 | 45 ± 9 |
|  | 5 | 148 ± 6 | 120 ± 6 | 263 ± 12 | 12 ± 0.5 | 29 ± 0.5 | 77 ± 2 | 45 ± 1 | 36 ± 7 | 57 ± 11 |
|  | 6 | 139 ± 7 | 107 ± 7 | 248 ± 9 | 12 ± 0.6 | 31 ± 1.8 | 74 ± 3 | 43 ± 2 | 53 ± 10 | 68 ± 9 |
| +/t | 0.25 | 153 ± 7 | 117 ± 8 | 300 ± 8 | 14 ± 0.3 | 32 ± 0.6 | 72 ± 2 | 38 ± 2 | 53 ± 5 | 82 ± 9 |
|  | 1 | 144 ± 7 | 109 ± 8 | 267 ± 9 | 13 ± 0.4 | 31 ± 0.5 | 71 ± 3 | 40 ± 2 | 61 ± 6 | 82 ± 10 |
|  | 2 | 128 ± 5 | 96 ± 8 | 236 ± 4 | 12 ± 0.3 | 32 ± 0.7 | 71 ± 4 | 40 ± 3 | 61 ± 9 | 89 ± 20 |
|  | 3 | 144 ± 6 | 105 ± 11 | 255 ± 7 | 13 ± 0.5 | 31 ± 1.0 | 70 ± 5 | 41 ± 4 | 69 ± 6 | 72 ± 14 |
|  | 4 | 131 ± 7 | 96 ± 8 | 242 ± 9 | 12 ± 0.3 | 30 ± 1.1 | 70 ± 4 | 39 ± 3 | 66 ± 9 | 95 ± 23 |
|  | 5 | 134 ± 3 | 94 ± 7 | 241 ± 5 | 13 ± 0.2 | 30 ± 0.6 | 68 ± 4 | 39 ± 3 | 72 ± 7 | 98 ± 19 |
|  | 6 | 120 ± 6 | 78 ± 7 | 219 ± 7 | 12 ± 0.5 | 29 ± 1.2 | 61 ± 3 | 34 ± 3 | 55 ± 8 | 85 ± 20 |

VAP = Average path velocity; VSL = Straight-line velocity; VCL = Curvilinear velocity; ALH = Amplitude of lateral head displacement; BCF = Beat cross frequency; STR = Straightness; LIN = Linearity; % motile = Percentage of sperm that were motile, averaged for 8 scans per male; Count = Average number of sperm measured in each scan.
